# A near-peer teaching program designed, developed and delivered exclusively by recent medical graduates for final year medical students sitting the final objective structured clinical examination (OSCE)

**DOI:** 10.1186/1472-6920-11-11

**Published:** 2011-03-17

**Authors:** Mustafa S Rashid, Oluwaseun Sobowale, David Gore

**Affiliations:** 1 Stockport NHS Foundation Trust, Poplar Grove, Stockport, Cheshire, UK; 2 Salford Royal NHS Foundation Trust, Salford, UK; 3 University Hospitals of Morecambe Bay NHS Foundation Trust, Lancaster, UK

## Abstract

**Background:**

The General Medical Council states that teaching doctors and students is important for the care of patients. Our aim was to deliver a structured teaching program to final year medical students, evaluate the efficacy of teaching given by junior doctors and review the pertinent literature.

**Methods:**

We developed a revision package for final year medical students sitting the Objective Structured Clinical Examination (OSCE). The package was created and delivered exclusively by recent medical graduates and consisted of lectures and small group seminars covering the core areas of medicine and surgery, with a focus on specific OSCE station examples. Students were asked to complete a feedback questionnaire during and immediately after the program.

**Results:**

One hundred and eighteen completed feedback questionnaires were analysed. All participants stated that the content covered was relevant to their revision. 73.2% stated that junior doctors delivered teaching that is comparable to that of consultant - led teaching. 97.9% stated the revision course had a positive influence on their learning.

**Conclusions:**

Our study showed that recent medical graduates are able to create and deliver a structured, formal revision program and provide a unique perspective to exam preparation that was very well received by our student cohort. The role of junior doctors teaching medical students in a formal structured environment is very valuable and should be encouraged.

## Background

Peer and near-peer learning are rapidly expanding areas of educational research across many disciplines [1,2]. Medicine is no exception and there are several descriptions of peer teaching in the medical literature [3]. This is perhaps not surprising considering the emphasis that is placed on teaching by the General Medical Council (GMC) in the UK and the international medical community [4,5]. Most medical professionals are now expected to teach students as an integral part of their role as doctor, indeed several authors in the field of medical education have highlighted that '*doctor*' in Latin means 'teacher' [5].

Numerous medical schools have a form of peer teaching integrated into their curriculum under the guise of problem-based learning PBL [6]. In medical literature the peer-assisted learning model has demonstrated benefits in many areas of medical teaching from socio-cultural diversity training to ultrasound image interpretation [7,8]. A key principle and benefit of peer-assisted learning is that peers can help other peers learn, consolidate and improve their knowledge in the process [7,9].

In 1988 Whitman introduced the term 'near-peer teaching' [10]. According to Bulte et al a near-peer tutor is 'a trainee one or more years senior to another trainee on the same level of medical education training' and a peer-tutor is one at the same level [5]. Rodrigues et al noted that many papers use the terms near-peer and peer-assisted learning interchangeably without reference to any seniority [11]. In 2007 Ten Cate and Durning surveyed the 2006 medical literature for reports of peer-teaching and although they found numerous descriptions they concluded many reports do not become full length journal articles [3]. Suggested reasons why so few are published in full include the often informal nature of peer-teaching and the lack of evaluation [12].

Ten Cate and Durning evaluated the reasons behind the introduction of peer teaching [3]. Ten of the studies they considered advocate peer teaching as a potential means of reducing teaching pressures on trained faculty staff [11-13]. Rodrigues et al further elaborate noting that the increasing number of medical students and constraints on doctors teaching (due to increased workload) is encouraging the development of such programmes [11].

A further rationale for peer teaching, and a possible explanation for the many positive outcomes is 'cognitive congruence', a concept described by Lockspeiser et al [14]. The close proximity of age and recent similar experiences of peer tutors provides an added benefit as near-peer teachers have a better appreciation of the knowledge held by junior peers and can therefore target teaching at an appropriate level [12]. This is supported by Rodrigues et al who comment that junior doctors are effective near peer teachers as their added experiences as a doctor, combined with their recent experience of medical school examinations, provides informed insight [11]. In addition Leeper et al suggest that cognitive congruence promotes a more relaxed learning environment [15]. Ten Cate and Durning discuss a similar 'comfortable and safe environment' for learning [3]. Peer teaching also provides a benefit to the teachers who can consolidate their knowledge as they prepare content, gain teaching experience, receive recognition for their teaching and often receive formal teacher training [5,11].

Given the lack of data regarding the effectiveness of near-peer teaching programs for a large student cohort we created a platform for junior doctors to gain experience in delivering teaching as well as developing educational resources and planning tutorials. Our primary aim was to evaluate a near-peer led revision program designed, developed, and delivered by recent medical graduates for a large number of final year medical students (n = 125) sitting their final objective structured clinical examination (OSCE). We aimed to show that junior doctors are effective near-peer tutors for medical students, providing a unique and valuable perspective to their OSCE preparation.

Junior doctors are well placed to teach students as they share recent experiences and are familiar with the assessment processes. Furthermore recent medical graduates acting as near-peer tutors should not be confined to the role of teaching delivery but can also effectively design, develop, and co-ordinate a revision program to a large number of students.

Our hypothesis is that:

• Junior Doctors can effectively design, develop, and co-ordinate a revision program to a large number of students

• Junior doctors can deliver teaching comparable to that of consultant led teaching.

## Methods

### Program design and development

The MasterOSCE medical and surgical finals OSCE revision program was created to address two important issues identified by the authors, all of whom are recent graduates and junior doctors. Firstly, many medical schools are continuously updating their examination format, in particular the final OSCE. This may prove a source of anxiety for many medical students [16-19]. Secondly, senior consultants often deliver the majority of revision teaching provided by medical schools. Although many junior doctors teach medical students, most of this is informal and undertaken impromptu on the wards [20].

We developed a revision specifically for final year medical students to assist preparation for their final OSCE at a medical school in the North West of England. Common topics encountered by foundation trainees were identified following a consultation process with junior doctors. A program was developed by the authors to include the common clinical scenarios identified in addition to topics thought to be difficult or 'tricky' by local students including safe prescribing skills, ethics and other speciality areas less frequently encountered such as ophthalmology and dermatology. Several modalities of teaching were incorporated in the program including lectures, small group seminars, videos, interactive modules and revision notes (made available online and in a course booklet). All teaching was prepared and delivered exclusively by junior doctors, who had recently completed a similar examination.

Broad content areas, that were considered core topics, were taught in a lecture format. These included cardiology, respiratory medicine, gastroenterology and neurology. Small group seminars were reserved for smaller topics as well as those areas thought to cause concern for medical students. This was to allow more opportunity to ask the tutor questions regarding these less familiar topics: ethics, communication skills, dermatology, radiograph interpretation, peripheral vascular and hernia examinations. A session named "Ask the panel" was reserved so that students could ask the near-peer tutors about OSCE technique, specific OSCE stations encountered or any concerns regarding their own examination preparation. Additional supplementary material was made available online in the form of revision notes. Furthermore, a course booklet was developed to provide students with written notes that complemented the topics taught in the lectures and small group seminars. The booklet also included material for the interactive modules placed within the program.

All three authors undertook the planning and co-ordination of the revision program as well as the production of all revision materials equally. This included creation of online revision notes, communication with near-peer tutors and recruitment of students. The development of content included in the lectures and small-group seminars was delegated to each near-peer tutor delivering that particular session and peer reviewed.

### Course structure

The revision course took place 4 months following the commencement of the final medical year and 6 weeks prior to the final OSCE. Online revision notes were accessible four months in advance of the course. Students received a course booklet on the first day of the course after registration. At this time a self-administered feedback questionnaire was provided to each participant. The two-day course followed a similar structure on both days. Lectures were delivered in the mornings, separated by coffee breaks. In the afternoon, a rotation system of five small group seminars was employed.

### Recruitment

Final year medical students attending a University in the North West of England were invited via email to participate in the program. Of the 457 students sitting the final OSCE examination, 125 were enrolled into the revision program on a first come, first served basis. Numbers were limited to 125 allowing for five small group seminars of 25 students each. Students were invited to attend through an online enrolment page. Three separate emails between 1 and 4 months prior to the commencement of the revision program were sent to all 457 final year medical students sitting the final OSCE examination. Enrolment was closed after 125 students were recruited.

Near-peer tutors were invited to attend an informal interview via email. There were no specific pre-requisites other than a passion to teach medical students and motivation to develop teaching skills. Six recent medical graduates expressed interest in being involved with developing and delivering the program. Each met with all three authors to discuss the aims of the study and to outline what was expected from their teaching. There was no formal interview process for recruiting near-peer tutors however an informal discussion with the authors occurred on an individual basis to outline the nature of the revision program and its principles. Topics were allocated based upon their personal preference, which was usually driven by career aspirations/areas of personal interest.

### Feedback

Prior to enrolment into the program all students were asked to read a document outlining how the data will be used to conduct research and electronically confirm they agree to these terms. This was in line with local ethical guidance and outlined how the data gathered from feedback questionnaires would be used and handled. Anonymised self-administered feedback questionnaires were provided to all participants at the time of registration.

The questionnaires contained four main sections including: pre-course questions, lectures and small group seminar feedback, free text comments and post-course feedback. Five point Likert scales were used for all questions. Students were asked about their confidence, expectations, preparation and previous teaching in the pre-course section. Feedback on lectures focused on clarity of aims/objectives, delivery, content, relevance and tutor knowledge. Students were asked to provide similar feedback on the small group seminars but also including organisation, efficiency, and usefulness of the session. Free text comments were encouraged following each day of the course. Finally a post course evaluation addressed what effect the program had on the students' confidence, preparation and expectations for their final OSCE. Specifically students were asked to compare teaching provided by junior doctors to traditional consultant-led teaching and whether they would recommend the program to future students.

All feedback questionnaires were collected at the end of the revision course therefore all feedback was provided at the time of or immediately after teaching delivery. Data was anonymous and securely stored.

## Results

One hundred and twenty-five final year medical students were recruited via emails Of the 125 attendees, 118 students (94.4%) completed self-administered feedback questionnaires.

### Pre-course questions

Responses to pre-course questions (n = 118) highlighted that 37.3% (n = 44) students did not feel confident about the forthcoming OSCE and a further 43.2% (n = 51) were neutral. Furthermore less than half (45.8%, n = 54) of students stated that they knew what to expect from the OSCE and 27.1% (n = 32) felt that the teaching received so far was adequate preparation for the OSCE. Students did not feel more confident about the clinical examination over the written papers or vice versa.

Free text comments were largely positive on both days of the course specifically relating to the relevance of content, quality of teaching and organisation of the program. The ask the panel session was found to be extremely useful for students, many of whom commented on how they felt better equipped to prepare for the OSCE following this session.

### Lectures feedback

One hundred and eighteen students gave 812 responses on seven lectures. Student's feedback towards the lectures delivered by near-peer tutors was very positive. The majority of students (88.1%, n = 715) found the aims and objectives were clear and identified early in the lecture. Furthermore, 85.2% (n = 692) thought the lectures were well delivered and 93.0% (n = 755) thought the near-peer tutor was knowledgeable in the topic taught. Students agreed or strongly agreed the content delivered in the lectures was relevant to the final OSCE in 92.4% (n = 750) of responses (mean Likert score 4.42/5.00; SD ± 1.16). Of the students responses to lectures 83.5% (n = 678) felt that having attended that session they feel more able to focus their examination preparation. Specifically 88.4% (n = 718) of students indicated that the content of the lectures would help preparation for the OSCE.

### Seminars feedback

Student feedback on the near-peer tutor led small group seminars was equally positive. Of the 1153 responses regarding small group seminars 91.4% (n = 1054) agreed or strongly agreed the aims and objectives of the sessions were clear whilst 96.4% (n = 1112) found the content covered was directly relevant to their OSCE preparation (mean Likert score 4.52/5.00; SD ± 1.26). Data (92.8%, n = 1070) also showed that students thought the content covered in the small group seminars would help them prepare for their OSCE. Students commented that they felt more confident on the topics taught in the small group seminars in 88.7% (n = 1023) of responses. 91.7% (n = 1057) of responses relating to the small group seminars stated that they were well organised and ran efficiently.

### Post-course feedback

All ninety-seven students that completed the post-course feedback questions stated that they felt better equipped to prepare for the OSCE. This 100% positive response rate was consistent regarding the relevance and usefulness of the content covered on the program (mean Likert score 4.48/5.00; SD ± 1.23). Interestingly 71 students (73.2%) agreed or strongly agreed that teaching delivered by the near-peer tutors was comparable to that of traditional consultant-led teaching. The majority of students (97.9%, n = 95) found that the MasterOSCE program had a positive influence on their OSCE preparation with 96.9% (n = 94) stating they would recommend the program to future medical students. 94.8% (n = 92) of students found the different teaching modalities and techniques incorporated into the revision program useful. In our cohort 96.9% (n = 94) of students stated that they know what to expect from their final OSCE having attending this program (Mean Likert score 4.38/5.00; SD ± 1.16). In addition, 95.9% (n = 93) felt more confident about their OSCE having attended our near-peer revision program (Mean Likert score 4.31; SD ± 1.15).

## Discussion

Several authors have compared peer tutors to medical faculty tutors and have found little or no effect in the quality of teaching or long term outcomes [8,12,13,21,22]. In a single blinded randomised controlled trial conducted at Oxford University Hughes et al compared peer led and expert led advanced cardiac resuscitation training [13]. They concluded that peer instructors could provide training to small groups comparable to that delivered by experts. Nestel et al acknowledged that students recognise and identify the benefits of having experienced medical teachers [12]. We agree that medical tutors have a significant role in delivering teaching to medical students however near-peers provide a unique perspective and approach that is commonly well received. Participants in most studies were volunteers and therefore they may be unrepresentative of the general student population as arguably the best motivated and most receptive students are most likely to participate [8,12].

In the majority of the studies conducted in this field peers or near peers teach small groups [5,12,13]. The number of students in a group in Hughes et al's randomised controlled trial was limited to 7 [13]. Each tutor lead a study group of only 4 students in the study by Rodriguez et al [11]. There is limited research into the effectiveness of near-peer led teaching programs for large groups of students. Our study included teaching in several sized groups ranging from twenty-five (small-group seminars) to 125 in a more traditional lecture-based format. We believe that any such program should include a variety of teaching modalities to keep the students engaged and motivated throughout. Furthermore, certain topics lend themselves well to being discussed in smaller groups such as ethics teaching. Our study has shown that students responded positively to teaching delivered by near-peer tutors using different modalities and group sizes provided that the content was relevant.

Bulte et al considered roles best suited for near-peer teachers [5]. Retrospective surveys of participants (learners and teachers) of near-peer teaching were evaluated. They identified that information provider; role model and facilitator were viewed as appropriate roles for near-peer teachers whereas planner and resource developer may be less suitable roles. This study did not consider the experience of the respondents, in terms of planning and resource management. It could therefore be the case that near-peer teachers rate these roles as less suitable simply because they lack experience. If near-peer tutors can be taught to provide information effectively it is plausible they can be taught to plan and manage resources. Our study showed that a program developed and delivered exclusively by near-peers could be beneficial to medical students preparing for an OSCE. It is important to note that external support is also incredibly useful, especially from experienced faculty staff. However we believe that such programs are well received by students when external influence on content is limited. Leeper et al describe an education module designed and presented by medical students for their peers, with faculty assistance and support [15]. The outcomes were positive and the module was described as 'inherently student orientated'. Students performed well when module content appeared in their final examination.

Rodrigues et al describe a near-pear teaching scheme, devised by junior doctors who developed prescribing and clinical examination sessions for medical students [11]. Medical students again fed back favourably and a positive effect was noted in a mock examination where attendees made significantly fewer prescribing errors.

Some resistance against near-peer teaching programs centre on lack of training and experience of near-peer tutors. There is limited research considering whether peer-teachers require extensive training. Knobe et al compared the teaching of musculoskeletal ultrasound by peer-teachers with experienced teachers and reported that there was no difference in student outcomes between the groups [8]. This study was limited to ultrasonography teaching and any extrapolation to other aspects of undergraduate medical education should be made with caution. However there is still no evidence to suggest that near-peer tutors without formal training provide teaching that is detrimental to students. Knobe et al also report that didactic training of the peer-teachers was not necessary. In a similar study Tolsgard et al compared teaching of catheterisation and intra-venous access and found that students taught by peers performed as well as experienced teachers (associate professors). Like many authors Tolsgard et al regard the training of peer-teachers as important [21,22]. We are keen to explore this in subsequent work.

A limitation common throughout current research, including this paper, is the selection of motivated peer teachers, with special interests in medical education. This was noted by Tang et al who commented that prospective tutors enrolled to enhance their skillset [7]. Consequently the results of these papers may reflect the benefit of peer assisted learning by well-motivated peer teachers. It could equally be said however that we should not expect near-peer tutors to have special interests early in their career. Our near-peer tutors were recruited based on an expressed interest to teach medical students. They too fall into the select cohort of enthusiastic and well-motivated near-peer tutors. Further research is required to identify early attributes in medical students that may develop into an interest in becoming near-peer tutors later in their training.

This limitation can also be applied to our sample cohort. By enrolling students on a first come first served basis our sample cohort is not fully representative of the entire final year medical student population as the most enthusiastic and diligent students are likely enrol in such programs earlier than less motivated students. Furthermore, some students feel that near-peer learning does not suit their learning style and that revision courses are not necessary in preparation for OSCE examinations. These students are therefore also not represented in our study by self-exclusion.

Despite our positive results it is important to recognise other limitations of this study. Firstly, the data gathered were entirely qualitative from self-administered questionnaires. Furthermore, questionnaires were provided at the beginning of the course and students were asked to complete the evaluation at the time of teaching. Such immediate feedback is likely to be favourable and therefore a repeat feedback request after a period of time could be employed in future. Later feedback was not sought due to logistic difficulties, as medical students undertake an overseas elective module soon after the final OSCE. Comparison to OSCE results would be a useful measurement that could be applied to future studies.

Another important consideration is that although this program produced good results and was well valued by final year medical students it may not be necessarily transferable to other situations. The success of this and any program relies on the motivation and enthusiasm of the near-peer tutors as well as the students who participate. Furthermore, our student population were approaching a final clinical examination and this program may have been successful solely because students often welcome any supplementary teaching.

The conditions for success in our revision program were the enthusiastic and dedicated recent graduates with personal knowledge of the examination system. A well-organised structure utilising appropriate teaching styles applied according to topics. And this inclusion of 'tricky' topics not thoroughly covered by traditional teaching. An important factor as to why near-peer revision programs such as this one succeed is regarding the students' expectations and confidence prior to sitting clinical examinations and how this is affected by attending near-peer teaching.

## Conclusions

This work has highlighted some interesting points within the field of medical education. We have demonstrated findings in keeping with the current research that teaching delivered by near-peer tutors was very well received by medical students. Students found that recent medical graduates provided a unique perspective to OSCE preparation. The teaching and advice offered by recent medical graduates to their undergraduate near-peers helped improve confidence and align expectations for OSCE preparation.

This study has demonstrated that a revision program can be designed, developed and delivered by recent medical graduates without external influences from senior colleagues. Near-peer teaching can arise from a process of development that does not necessarily involve senior consultants and professors. It is important however that teaching delivered by near-peers does not have potentially negative effects on medical students examination preparation and therefore senior support is very beneficial in such programs. Furthermore this study provides evidence that junior doctors can successfully effectively design, develop, and co-ordinate a revision program to a large number of students. We therefore support the use of junior doctors as near-peer tutors in variety of roles aside from teaching delivery including program development and organisation.

## Competing interests

The authors declare that although our project is not for profit our revision program does include a subscription charge to cover the administration and running costs.

## Authors' contributions

MSR, OS and DG contributed equally to this report. This included development of the resources, delivery of the program, gathering and analysis of feedback data, and drafting the manuscript. All authors read and approved the final manuscript.

## Pre-publication history

The pre-publication history for this paper can be accessed here:

http://www.biomedcentral.com/1472-6920/11/11/prepub
